# Self-report measures of executive functioning are a determinant of academic performance in first-year students at a university of applied sciences

**DOI:** 10.3389/fpsyg.2015.01131

**Published:** 2015-08-05

**Authors:** Maria A. E. Baars, Marije Nije Bijvank, Geertje H. Tonnaer, Jelle Jolles

**Affiliations:** ^1^Department of Educational Neuroscience, Faculty of Psychology and Education, LEARN! Institute, VU University AmsterdamAmsterdam, Netherlands; ^2^Department of Social Work, Faculty of Health, Behavior and Society, HAN University of Applied SciencesNijmegen, Netherlands; ^3^Saxion University of Applied SciencesDeventer, Netherlands

**Keywords:** executive functions, academic performance, determinants, individual differences, intervention design

## Abstract

Recent studies in late adolescents (age 17+) show that brain development may proceed till around the 25th year of age. This implies that study performance in higher education could be dependent upon the stage of brain maturation and neuropsychological development. Individual differences in development of neuropsychological skills may thus have a substantial influence on the outcome of the educational process. This hypothesis was evaluated in a large survey of 1760 first-year students at a University of Applied Sciences, of which 1332 are included in the current analyses. This was because of their fit within the age range we pre-set (17–20 years' old at start of studies). Student characteristics and three behavioral ratings of executive functioning (EF) were evaluated with regard to their influence on academic performance. Self-report measures were used: self-reported attention, planning, and self-control and self-monitoring. Results showed that students with better self-reported EF at the start of the first year of their studies obtained more study credits at the end of that year than students with a lower EF self-rating. The correlation between self-control and self-monitoring on the one hand, and study progress on the other, appeared to differ for male and female students and to be influenced by the level of prior education. The results of this large-scale study could have practical relevance. The profound individual differences between students may at least partly be a consequence of their stage of development as an adolescent. Students who show lower levels of attention control, planning, and self-control/self-monitoring can be expected to have a problem in study planning and study progress monitoring and hence study progress. The findings imply that interventions directed at the training of these (executive) functions should be developed and used in higher education in order to improve academic achievement, learning attitude, and motivation.

## Introduction

For most students, the transition from secondary education to higher education is characterized by a mix of excitement, high expectations and anxious distress in a context of environmental change (Lowe and Cook, 2003; Casey et al., 2010). The professional or academic learning environment is different from the learning environment in secondary education in both learning material and procedures. In addition, many young students are considered to be in the phase of “late adolescence” (Veroude et al., 2013a), a period described by others as the “phase of emerging adulthood” (Arnett, 2000). This period, referring to persons aged 18–25, is not only characterized by a shift in educational environment but also by major changes in physical development (Dahl, 2004), as well as psychosocial functioning and “personal growth” (Steinberg and Morris, 2001). Late adolescents and young adults leave home, make new friends and lose others, establish new social networks and work to obtain financial independence. They thus experience profound changes in the physical, psychological, and social domain.

Major changes in neuropsychological functioning, feelings, social cognitions, and behavior appear to be supported by maturation of underlying brain networks and structures (Steinberg and Morris, 2001; Casey et al., 2008). Several studies in structural and functional brain imaging have shown that core areas in the prefrontal cortex and their connections to many other structures in the brain are still in a process of maturation in adolescents and emerging adults (aged 18–23 years) (Shaw et al., 2008; Giedd and Rapoport, 2010). Other studies showed that particular neuropsychological functions, described also as “executive functions” (EF), are still under development over the long period of adolescence matching the stage of brain maturation (Huizinga et al., 2006; Best et al., 2011). Recent, work from our research group has added new findings to this line of evidence. In an fMRI study into brain maturation and cognitive control (Veroude et al., 2013b), late adolescents (aged 18–19 years) were compared to young adults (aged 23–25 years) with regard to their performance on computerized neurocognitive tests and emotional Stroop tasks. The study showed that the neural bases for self-control (measured with a cognitive and emotional Stroop task) changed between late adolescence and young adulthood. Another study showed that adolescence is associated with a continuous increase in the ability to cope with delayed gratification (Lee et al., 2013). Working hard for good test results in due time challenges students' perception of investment vs. reward, since they are not fully equipped to cope with delayed gratification yet (Christakou et al., 2011; Lee et al., 2013).

The development of decision-making processes that rely on EF characterize the transition from secondary education to a learning environment in higher education. In adolescence, applying self-control, and self-monitoring has been shown to be particularly difficult in an emotional context (Casey et al., 2011). The new academic environment challenges students' self-control and self-monitoring skills since more academic autonomy is expected than in secondary education, while combining important life events with controlling the social relations and the emotional aspects involved. Potential lack of self-control may negatively affect study progress, especially in first-year students. We know from previous studies that lack of study progress in the first year of higher education is a good indicator of college-dropout later on (Lowe and Cook, 2003; Arulampalam et al., 2004; HBO-Raad, 2011) and that students with EF deficits achieve lower GPAs than students without EF deficits (Knouse et al., 2014). Dropout has many short- and long-term negative consequences, both for students and for universities, and is a major problem for many university departments. For example, in the Netherlands, 22% of first-year students at Universities of Applied Science do not graduate within 8 years after starting their 4-year study program, and on average 16% of all students drop out of their study within their first year (HBO-Raad, 2011). Therefore, studying self-control and its effect on academic performance of first-year students will increase insight into the processes underlying study success. We expect a better understanding of this relation to reveal possibilities for interventions aimed specifically at students at risk of slow study progress.

Consequently, in this epidemiological study, we aimed to investigate the combined influence of demographic factors and particular aspects of executive functioning on academic performance in first-year students. Self-perceived attention, planning and self-regulation/self-monitoring were measured with a rating scale which shares an approach taken by the well-known BRIEF (Behavioral Rating Inventory of Executive Functioning, Gioia et al., 2000). We expected the level of these self-perceived executive functions to be predictive of study progress, with lower levels of EF resulting in less study credits obtained. We measured EF by means of self-report in a validated questionnaire (Van der Elst et al., 2012). The self-report questionnaire was used instead of standard neuropsychological tests since we are interested in behavior, self-insight and those executive functions that cannot be measured by objective cognitive tests. These non-cognitive functions are important for functioning in daily life and are also part of the BRIEF mentioned above (Gioia et al., 2000). Our study was aimed at establishing whether or not there is a relation with study progress. Our study was not intended to evaluate brain-behavior mechanisms involved in the cognitive aspects of EF (Diamond, 2013). In addition, rating scales have been shown to out-perform EF tests in predicting outcomes in college students, (Wingo et al., 2013) and the ecological validity of EF tests is often poor (Barkley and Murphy, 2011) Our self-report questionnaire focused on measuring the level of: (1) attention; (2) planning; and, (3) self-control and self-monitoring. We expected all three measures to be predictive of first-year study success. In addition, age, sex, and level of prior education were considered in this study, since these may influence study success and the association between executive functions and study success.

## Materials and methods

### Participants and background characteristics

Data collection for this study was part of a large survey conducted in first-year students at the Hospitality Business School (HBS) of Saxion University of Applied Sciences in Deventer, the Netherlands. Self-report data on demographics, study behavior, executive functioning, and lifestyle were combined with data on objective study performance, retrieved from the student registry. The self-report data were gathered in the first period of the first semester students were enrolled, the data on objective study performance were gathered throughout the first year of studying, starting with the exams after the first period. Therefore students did not have any information on their objective study performance at the time they completed the survey. The longitudinal design of this study makes it a proper source of data for investigating effects of student characteristics on academic performance over time.

The in-classroom survey was performed by asking students to voluntarily participate in filling out a questionnaire. The teachers were instructed to hand out the forms and gather them after students had time to fill them in (approximately 20 min per questionnaire). The students were informed that no personalized data would be used in the analyses and that no personalized results would be obtained, since all data are assembled on group level.

Of a total of 1760 students who started their studies between 2010 and 2013, 1320 are included in the current analyses because of their fit within the age range we pre-set (17–20 years' old at start of studies). In this sample of students, levels of prior education included: intermediate vocational education (1); higher general secondary education (2); and pre-university education (3). Descriptive statistics are shown in Table 1. The majority (70.2%) of the first-year HBS students included in the survey study were female; on average they were 19 years old at the start of their studies; and higher general secondary education was the most prevalent type of prior education (60.8%). Crosstabs analyses showed no interactions between age or sex and level of education, or between age and sex.

**Table 1:** **Sample characteristics**.

	**Mean**	**SD**	**Range**
Sex (% female)	70.2		
**LEVEL OF EDUCATION (% PER LEVEL)**			
Secondary vocational education	25.8		
Higher general secondary education	60.8		
Pre-university education	13.4		
Age	18.9	1.3	17–21

### Instruments and procedure

First-year study success, defined as study progress, was measured by the total number of credits according to the European Credit Transfer and Accumulation System (ECTS EU, 2015) that were achieved on first exam attempts in the first year after study enrolment. A single ECTS represents a study load of 25–30 h. The maximum amount of ECTS to be attained in year one was 60 for students who had been enrolled the entire year (total ECTS are calculated relative to the total amount that a student could have received while being enrolled).

#### Amsterdam executive function inventory

The Amsterdam Executive Function Inventory (AEFI), (Van der Elst et al., 2012) was developed to measure executive functioning (EF) in adolescents by means of a short self-report questionnaire consisting of 13 questions. The responses for the AEFI items were presented on a 3-point Likert scale with the choice options 1 = “not true,” 2 = “partly true,” and 3 = “true.” Validity and reliability of this questionnaire were evaluated in a large sample of adolescents aged 15–18 years and described elsewhere (Van der Elst et al., 2012). A slightly modified version of the AEFI was used to identify EF in the current study. Items were adapted to match the age and life stage of the student group included in this study (see Table A1 in Appendix for original and adapted AEFI items). The original scale included 13 items and the revised version had 10 items. The adapted version has higher levels of reliability than the original scale (Table 2) for all three subscales. Standardized factor loadings were also higher than those of the original scale. In our revised version, the third subscale is focused solely on planning, while the original scale combined planning and aspects of initiative taking. This choice is based on results of factor and reliability analyses.

**Table 2:** **Factor and reliability analyses for the adapted AEFI scale**.

**Variable**	**Factor loadings AEFI**	**Factor loadings adapted AEFI**	**Cronbach's α AEFI**	**Cronbach's α adapted AEFI**
Attention	0.69	0.81	0.64	0.78
Planning (& initiative)	0.54	0.74	0.60	0.65
Self-control and self-monitoring	0.60	0.71	0.65	0.69

### Data analysis

Linear regression analyses were used, using the backward stepwise procedure, to test for associations between background characteristics and the separate EF measures with study progress. Analyses of interactions between background variables and EF were performed using regression analyses as well. Also, the association between a total adjusted AEFI score as EF factor including the three subscales, was tested in linear regression analysis. To study the effects of the level of prior education, two dummy variables were created, with intermediate vocational education as reference category to higher general secondary education (LE Dum 1), and to pre-university education (LE Dum 2). All statistical analyses are performed using IBM SPSS Statistics for Windows, Version 20.0. Armonk, NY: IBM Corp.

## Results

Descriptive analyses indicated that mean AEFI score for the students included in the study was 32 (SD 5.0). An average of 41.8 ECTS were obtained during the first year of studies (SD 17.8) (Table 3).

**Table 3:** **Descriptive statistics for EF measures and study progress**.

	**Mean**	**SD**	**Range**
Total AEFI score	32	5.0	15–49
Study credits	41.8	17.8	0–96

Regression analyses showed that total adjusted AEFI score (β 0.15, *p* < 0.000) was positively associated with study progress, as measured by ECTS credits attained in the first year of studying (Table 4). Higher levels of attention (β 0.13, *p* < 0.000), planning (β 0.15, *p* < 0.000), and self-control (β 0.06, *p* < 0.05), were all associated with more study progress. There is a small difference in effect between male and female students for self-control and self-monitoring, favoring the female students (β 0.15, *p* < 0.000). The positive association between cognitive control and study progress was stronger for female students. In addition, level of prior education was positively associated with study progress–more highly educated students performed better than students with a lower level of prior education (β 0.18/0.24 *p* < 0.000). Sex showed a small separate effect on study credits as well. Female students outperformed males (β 0.12, *p* < 0.000). No interaction effects of education level and any of the AEFI scores were found, and the backward regression procedure implemented in the analyses caused age to be excluded from the final model.

**Table 4:** **Results of separate regression analyses of EF factors in association to background characteristics and with study progress**.

**Variable**	**B**	**SE(B)**	**T**	**Sig. (p)**	R^2^^*^
Sex	4.70	1.24	3.80	0.000	
LE Dum 1	6.60	1.37	4.81	0.000	
LE Dum 2	11.9	1.88	6.39	0.000	
Attention	1.04	0.25	4.11	0.000	0.08
Planning	1.31	0.27	4.82	0.000	0.09
Self-control and self-monitoring	0.42	0.21	2.03	0.04	0.07
Self-control and self-monitoring^*^Sex	0.36	0.07	4.91	0.000	0.07
EF factor total AEFI score	0.53	0.11	4.72	0.000	0.09

* *The R^2^ measures reported refer to the regression models including the separate EF factors*.

## Discussion

The main results of our analyses showed that the level of attention, planning, and self-control and self-monitoring of students included in this sample were predictive of their first-year study progress. This finding confirms our hypothesis that variance in the level of EF of students in part determines their study success. It is in line with results from recent studies into the development of EF, which show that these functions are still developing in late adolescence and emerging adulthood (Blakemore and Choudhury, 2006; Crone and Ridderinkhof, 2011; Veroude et al., 2013a,b), and also with results from a very recent study indicating that EF deficits predict the lack of study success in college students (Knouse et al., 2014). Our results provide further evidence that the capacity to anticipate and foresee the consequences of one's actions in the medium and long run is still developing even in young adults (Christakou et al., 2011; Lee et al., 2013). Our main results are in line with studies that indicate that a lack of EF maturation will make it challenging for students to deal with the profound changes in the physical, psychological, and social domain that are involved in the transition from secondary to higher education (Lowe and Cook, 2003; Casey et al., 2010).

Our additional analyses showed that the effect of level of EF on study performance is different for males and females. We know from recent literature that school performance in boys raises major concerns (Moreau, 2011). This study provides evidence that in higher education, male students may deserve specific attention—and possibly dedicated educational/pedagogical interventions—as was also indicated by previous work on EF differences in boys and girls attending high school (Dekker et al., 2013). We found the effect of lack of self-control and self-monitoring to be larger in males than in females, while there was no age difference between the two sexes. In line with previous studies indicating a time lag in brain development in boys, we found that this lag persists well into adulthood and affects study progress in higher education (De Bellis et al., 2001; Lenroot et al., 2007; Giedd, 2008).

Interestingly, we did not find an interaction between education level and EF with regard to study progress. This is in part explained by the age and education level distribution in our sample. The students with the lowest level of prior education were on average older than the students with the two higher levels of prior education. This may be explained by the fact that the two higher levels are types of high school, while the lowest level is a type of vocational education that allows entrance to the lowest level of high school (and so students who start out at the lowest level of high school need to finish both in order to be able to enroll in a University of Applied Science). Another reason may be that EF maturation is simply a stronger predictor of study progress compared to level of prior education, therefore overruling the effect of level of prior education when combined in an interaction analysis. The notion expressed here is a hypothesis, which in fact states that a person who is somewhat older (e.g., 2 years) may in fact have acquired additional skills because of the fact that he/she has been challenged for 2 more years, and that these experiences can be more important than the experiences acquired in school in the preceding period. This would explain the effects found in the interaction analysis.

The effect of EF on study progress established by our findings provides us with grounds for interventions aimed at improving the study success of first-year academic students. We know from previous studies (Lowe and Cook, 2003; Arulampalam et al., 2004) that dropout and lack of study progress are important issues in first-year academic students, warranting clearly targeted intervention. Combined with our results, this has implications for student-counseling facilities and -policy within universities. Individual differences with regard to the level of development of EF cause some students to be well adapted for the challenges which academia poses, while others need guidance and help, to make their transition to academic life more successful. The simple fact that a large proportion of first-year students do not yet have adequate planning or self-control and self-monitoring skills makes them vulnerable to slower study progress and possibly even drop-out. Targeted interventions, implemented early on in the first year of studying may help diminish this vulnerability and train first-year students in the basic skills they need to develop in order to succeed in higher education. These interventions should not only focus on training study-related skills, such as summarizing texts and planning study activities, but also on the personal growth that is necessary to learn how to combine the demands made by student life with a successful academic performance. These so-called non-cognitive skills that influence academic performance are also important to take into account (Morrison Gutman and Schoon, 2013) Gaining insight into self-monitoring issues, lack of self-control, and issues such as peer pressure and choosing your own path, will help students to better understand the challenges they face and may help them cope with these challenges. It is known from clinical studies of neuropsychological training aimed at improving executive functions that both metacognition (Hoogenhout et al., 2012), and executive functioning (Valentijn et al., 2005; In de Braek et al., 2012) can benefit from psycho-education and goal management training. These principles may be used to develop interventions aimed at improving planning, attention, and cognitive control in first-year students, as they have also shown relevance in middle school groups (Brannigan, 2006). Within Saxion University of Applied Sciences we developed a short course (4 meetings of 2 h) targeted at improving self-control and self-monitoring in students by first enlarging their knowledge of development of executive functions and the importance of these functions on academic performance. The second part of the course was aimed at training students in setting realistic goals for themselves regarding studying and aiming to improve academic results, but also regarding healthy behavior that is known to influence school functioning (Tremblay et al., 2011). The first pilot study of this course is being performed this academic year.

In order to interpret the results presented here, some challenging issues should be addressed and reflected upon. First of all, this study was performed at a university of applied sciences and it included 1760 first-year students at the Hospitality Business School, of which 1170 are included in the current analyses because of their fit within the age range we pre-set (17–20 years' old at start of studies). As far as we know, this is the first study into the direct association between EF and academic performance in university students that shows the importance of taking into account executive functions when evaluating study success. Hence, the results require confirmation in other samples.

Secondly, the self-report nature of the EF measure included in this study requires explanation. The use of a self-report questionnaire fit the goal of this study, since the AEFI consists of items focusing on everyday executive functioning, as experienced in a classroom or academic setting. Rating scales have been shown to out-perform EF tests in predicting outcomes in college students (Wingo et al., 2013) and the ecological validity of EF tests is often poor (Barkley and Murphy, 2011).

Thirdly, at the time of the current analyses no data on long-term study progress were available. However, data are longitudinal in the sense that EF functioning was measured at the beginning of students' first year and that their study performance was measured during the entire first year. One of our future aims is to follow the academic performance of these students throughout their academic career, and to follow their EF development as well.

Finally, in our design, we chose not to incorporate age as a factor in our final model. Hence, we decided only to include students within a narrow age range in our analyses (Mean age = 19.0, range 17–20 years, *SD* = 1.3). The choice to include only students within this restricted age range was made to reduce variance caused by age, in order to focus evaluation on other factors, such as sex, education level, and level of executive functioning.

In conclusion, early identification of specific student groups based on the stage of development of their cognitive functions is important in order to specifically aim interventions that might help academic growth and study progress at the right target group. Since non-cognitive functions such as motivation as crucial to academic achievement as the executive functions, students should be trained in the development of these functions throughout their academic careers, starting in primary school and continuing at university. This study has identified students with a lower level of executive functions as a target group for interventions, and has also shown that male-female differences should be considered, as well as differences in education level.

### Conflict of interest statement

The authors declare that the research was conducted in the absence of any commercial or financial relationships that could be construed as a potential conflict of interest.

## Appendix

**TABLE A1:** **AEFI items: original and adapted version**.

**Original**	**Adapted**
**Attention**	**Attention**
I am not able to focus on the same topic for a long period of time	I am not able to focus on the same topic for a long period of time
I am easily distracted	I am easily distracted
My thoughts easily wander	My thoughts easily wander
**Planning and initiative**	**Planning**
I can make fast decisions (e.g., in lessons)	I am well organized. For example, I am good at planning what I need to do during a day
I am well organized. For example, I am good at planning what I need to do during a day	I am chaotic or disorganized
It is easy for me to come up with a different solution if I get stuck whensolving a problem	My work is very tidy
I am full of new ideas	
I am curious, I want to know how things work	
**Self-control and self-monitoring**	**Self-control and self-monitoring**
I often react too fast. I've done or said something before it is my turn	I often react too fast. I've done or said something before it is my turn
It is difficult for me to sit still	Compared to others, I talk a lot
It takes a lot of effort for me to remember things	I do not consider the consequences before I act
I often forget what I have done yesterday	I am a blabbermouth
I often lose things	
